# Herbal Extracts Incorporated into Shortbread Cookies: Impact on Color and Fat Quality of the Cookies

**DOI:** 10.3390/biom9120858

**Published:** 2019-12-11

**Authors:** Mariola Kozlowska, Anna Zbikowska, Katarzyna Marciniak-Lukasiak, Malgorzata Kowalska

**Affiliations:** 1 Department of Chemistry, Institute of Food Sciences, Warsaw University of Life Sciences-SGGW (WULS-SGGW), 02-776 Warsaw, Poland; 2 Department of Food Technology and Assessment, Institute of Food Sciences, Warsaw University of Life Sciences-SGGW (WULS-SGGW), 02-772 Warsaw, Poland; anna_zbikowska@sggw.pl (A.Z.); katarzyna_marciniak_lukasiak@sggw.pl (K.M.-L.); 3 Department of Chemistry and Organic Materials, Faculty of Chemical Engineering and Commodity Science, University of Technology and Humanities in Radom, 26-600 Radom, Poland; mkowalska7@vp.pl

**Keywords:** pastry goods, plant material, sensory properties, peroxide value, *p*-anisidine value, DPPH assay

## Abstract

This study aimed at determining the effect of aqueous ethanolic extracts from lemon balm, hyssop and nettle, and butylated hydroxyanisole (BHA) on properties of shortbread cookies. This was achieved by instrumental measurements of color and sensory properties of the cookies directly after baking and by determination of peroxide (PV) and *p*-anisidine (*p*-AnV) values, and specific extinction coefficients (K_232_ and K_268_ values) for fat extracted from the cookies stored for 3 months at room temperature. Increase of the herbal extracts’ concentration from 0.02% to 0.2% in the cookies caused a reduction of L* (the brightness) and a* values (the red coordinate), while b* values (the yellow coordinate) increased when the cookies were enriched with lemon balm and nettle extracts. Among the cookies studied, those prepared with BHA and 0.1 and 0.2% addition of lemon balm extracts were characterized by the highest scores for aroma, taste, and overall acceptability. Incorporation of BHA and 0.02% hyssop extract into the cookies caused a decrease of PV values (the peroxide value) for fat extracted from the cookies after 3 months of their storage compared to a (control) sample without additives and produced the lowest K_232_ values. Changes in the *p*-AnV values for the fat samples studied occurred gradually and slowly during the storage and the obtained values were lower compared to the control sample. All of the studied fat samples also showed a higher ability to scavenge DPPH radicals than the control sample. Considering both PV and *p*-AnV values as indicators of fat oxidation, BHA protected fat extracted from cookies against oxidation better than the herbal extracts used.

## 1. Introduction

Shortbread cookies are pastry goods characterized by density and brittleness due to high quantities of fat rich in saturated fatty acids (SFA), especially myristic, palmitic, and stearic acids [1]. However, high consumption of SFA is associated with an increased risk of cardiovascular diseases. For this reason, much attention is focused on the improvement of their sensory and nutritional profiles through preparation of low-fat cookies using emulsifiers, interesterified shortenings, or some vegetable oils rich in monounsaturated fatty acids (MUFA) and polyunsaturated fatty acids (PUFA) [2,3]. The presence of oleic, linoleic, and linolenic acids in the chemical composition of vegetable oils may have a positive effect on health and may also be conducive to oxidation of a double bound they contain and thus, cause development of off-flavor and odors unacceptable to consumers [4]. Therefore, application of plant materials as sources of many active antioxidants instead of synthetic antioxidants can be an interesting alternative in preventing the oxidation of fat contained in shortcrust pastry goods, and also because of their beneficial effects on human health. Spices and herbs, especially from the Lamiaceae family like rosemary, thyme, and marjoram, are well-known for their antioxidant activity and as excellent sources of phenolic compounds that contribute to prevention of lipid oxidation and to improving quality and nutritional value of foods [5,6].

Lemon balm (*Melissa officinalis* L.) and hyssop (*Hyssopus officinalis* L.) are also perennial herbs belonging to the Lamiacea family, cultivated worldwide. Lemon balm is an important medicinal plant and a versatile culinary herb which can be used to flavor different products owing to its particular taste. It is very useful for treating nervous agitation and for promoting sleep, and it ameliorates functional gastrointestinal complaints [7]. Lemon balm is also used to treat herpes, gout, sores, insect bites, skin diseases, and as an insect repellent [8]. It can be added to salads, butters, cheeses, ice cream, jams, sauces, or cookies. Lemon balm leaves, flowers, and branches are a source of volatile oils, glycosides of alcoholic triterpene acids, and phenolic compounds such as phenolic acids and flavonoids [7]. The content of volatile oils in lemon balm is quite low compared with other members of the Lamiacea family and because of this, the price of the volatile oil is very high in the market. The volatile oil from lemon balm may be used as an antivirus, antimicrobial, and antifungal agent, effective against a series of human cancer cell lines and mouse cell line and as an antioxidant [9]. In turn, lemon balm aqueous and alcoholic extracts are rich in phenolic acids such as rosmarinic, caffeic, chlorogenic, and ferulic acids. Presence of these compounds in extracts may affect their antibacterial, antiviral, and antioxidant activities [10,11]. Lemon balm extract may be helpful in terms of preventing lipid deterioration in sausages and pork meat patties and in stabilization of long-chain fatty acids of algae oil [12].

Hyssop is an important medicinal plant used in tea blends for cough relief, antispasmodic, stomachic, and antifungal effects [13]. Its essential oil, rich in pinocamphore, *β*-pinene, myrtenol, linalool, methyleugenol, and limonene, is used in the food, pharmaceutical, and cosmetic industries [14,15]. Like other aromatic plants from the Lamiaceae family, hyssop extracts contain phenolic compounds, especially chlorogenic, protocatechuic, ferulic, syringic, caffeic, and rosmarinic acids [16]. The positive correlation between phenolic acids and flavonoids present in hyssop extract may indicate its significant antioxidant activity [17]. Among studied plant extracts, the ethanolic extract of *Hyssopus officinalis* has shown a good antioxidant activity but was characterized by the lowest total polyphenols and flavonoids content [18]. The inhibitory activity of the hyssop extract during autooxidation of lard was also observed [19]. Hyssop extracts also inhibited lipid oxidation and degradation of heme pigments after meat cooking and storage [20].

Stinging nettle (*Urtica dioica* L.) is a medicinal and edible plant, and fodder for animals. It is used in pharmaceuticals, cosmetics, and foodstuffs. Dried nettle leaf preparations are known to alleviate symptoms associated with allergic rhinitis and are a sustainable source of textile fiber [21]. Nettle leaves extract is used as a diuretic, in infections of urinary tract, as an adjuvant remedy in rheumatoid arthritis, in renal calculi and gout [22]. Nettle leaves are a good source of ascorbic acid and pro-vitamin A, essential amino acids, carbohydrates, fatty acids, carotenoids, and several minerals [23]. Stinging nettle could be used as a good natural, low-cost green pigment source, a vegetable in juice tea, and an ingredient in many dishes. Compared to barley and wheat flour, nettle flour has a much higher level of tannin, total polyphenols and content of protein, crude fiber, ash, and calcium and has a low glycemic index [24]. Use of natural antioxidants from water nettle extract as functional ingredients significantly reduced lipid oxidation, increased color stability during storage, and improved functionality of the final product [24]. Moreover, it also showed antimicrobial activity against some microorganisms, antiulcer activity against ethanol-induced ulcerogenesis, and analgesic effect on acetic acid-induced stretching [25]. The capacity of nettle extracts to improve oxidative stability of brined anchovies has been also reported [26].

The present study was undertaken to prepare shortbread cookies by partially replacing palm fat with cold-pressed walnut oil supplemented with aqueous ethanolic extracts from lemon balm, hyssop, and nettle. Color intensity and selected sensory properties of the shortbread cookies after baking were determined. Then, fat stability extracted from the cookies during their storage at room temperature by determining the peroxide, *p*-anisidine values, specific UV extinctions (K value), and the capacity of fat samples containing herbal extracts to scavenge DPPH radicals were evaluated.

## 2. Materials and Methods

### 2.1. Materials and Chemicals

Aqueous ethanolic extracts from plant materials, such as dried leaves of lemon balm (*Melissa officinalis* L.), hyssop (*Hyssopus officinalis* L.), and nettle (*Urtica dioica* L.), were used as fortification agents, were prepared in advance according to Kozłowska et al. [27]. Ingredients for making shortbread cookies were purchased in a local market (Warsaw, Poland). These were palm fat “Akofect LT M53” (AarhusKarlshamn, Malmo, Sweden), cold-pressed walnut oil “ULEI” (PROMET-T SA, Moldova), wheat flour type 480 “Szymanowska” (Polskie Młyny, Warsaw, Poland), sugar (Pfeifer & Langen Marketing, Poznań, Poland), and eggs. All the solvents (ethanol, methanol, diethyl ether, chloroform, *n*-hexane) and reagents (acetic acid, potassium hydroxide, potassium iodide, potassium chloride, sodium thiosulfate, starch soluble, phenolphthalein, anhydrous magnesium sulphate) used were analytical grade and were purchased from Avantor Performance Materials Poland (Gliwice, Poland). Butylated hydroxyanisole (BHA), 2,2-diphenyl-1-picrylhydrazyl (DPPH) and Trolox ((±)-6-hydroxy-2,5,7,8-tetramethylchromane-2-carboxylic acid) were obtained from Sigma-Aldrich (St. Louis, MO, USA). A certified fatty acids methyl ester (FAME) reference standard mixture (37 fatty acids from C4 to C24) was from Supelco (Bellefonte, PA, USA).

### 2.2. Shortbread Cookies Preparation

Dry ingredients comprising twice-sieved wheat flour (300 g) and sugar (100 g) were mixed using a spoon. Then eggs (50 g), whipped at medium speed using a kitchen mixer (Multiquick, Braun, Germany), were added and the mixture was blended. Fat was added (200 g) and blended for 1 min using Braun K650 Multiquick kitchen machine food processor (Germany). Then, the dough was kneaded by hand to combine the ingredients and placed in a refrigerator to cool at 4 °C (about 1h). The dough was rolled out to a thickness of 3 mm, cut into cookie shapes (5 cm diameter), placed on an aluminum tray and baked at 160 °C for 25 min in Unox convection oven (model XBC, Vigodarzere, Italy). The shortbread cookies were cooled at room temperature, packed in plastic pouches (PEHD) (every type of the cookies in a separate bag), and stored without access of light at room temperature for 3 months. They were taken out after 0, 1, 2, and 3 months of storage and used for further studies.

10 variants of fat were used in preparation of ten formulations of the shortbread cookies. For this purpose, palm fat (110 g) was weighed into flasks and dissolved in a water bath at 40 °C. Then cold-pressed walnut oil (90 g) and aqueous ethanolic extracts from lemon balm, hyssop and nettle at 3 different levels (0.02%, 0.1%, 0.2%), and synthetic antioxidant (BHA 0.02%) were added. The fat mixture was stirred using a kitchen mixer (Multiquick, Braun, Frankfurt, Germany) for 1 min and cooled in a refrigerator at 4 °C. Control samples contained fat (palm oil and cold-pressed walnut oil) without addition of antioxidants.

### 2.3. Fatty Acid Analysis

The fatty acid composition of the fat samples used in the preparation of the control samples was analyzed by gas chromatography (GC) after derivatization to fatty acid methyl esters with a 2 M methanolic solution of potassium hydroxide according to ISO 12966-2:2017 standard method [28]. A Shimadzu GC-17A (Kyoto, Japan) gas chromatograph equipped with a flame ionization detector (FID) and a BPX-70 capillary column (length 30 m, i.d. 0.22 mm, film thickness 0.25 µm; Melbourne, Australia) were used. The analysis was performed with nitrogen (1.0 mL/min) as a carrier gas at the following temperature program: 60 °C held for 1 min, after which the temperature was increased to 170 °C at a rate of 10 °C/min and from 170 to 230 °C at a rate of 3 °C/min. The temperature was kept at 230 °C for the subsequent 15 min. The injector (split ratio of 100:1) and detector temperatures were set at 225 °C and 250 °C, respectively. Individual fatty acids were identified by comparing their retention times with a certified fatty acid methyl esters mix and quantified as a percentage of the total fatty acids.

### 2.4. Color Measurement

The color intensity of the shortbread cookies with and without the addition of different concentrations of aqueous ethanolic plant extracts and BHA was determined after baking using a tristimulus reflectance colorimeter (Minolta CM-3600d, Konica Minolta Sensing, Inc., Tokyo, Japan). It was expressed as L*, a* and b*, where L* represents lightness of color (value 100) or blackness (value 0), a* represents red (positive value) or green (negative value), and b* defines the proportion of yellow (positive value) or blue (negative value). The final result was the arithmetic mean of 12 measurements.

### 2.5. Sensory Evaluation

The sensory properties of the shortbread cookies samples without and with different amounts of plant extracts and BHA such as aroma, taste and overall acceptability were evaluated by ten panel members from the Food Science and Nutrition Department. The samples (each weighing around 5 g) were served to the panelists on paper plates with water to cleanse and neutralize their palates 2 h after baking. All the samples were presented with three digit codes in a randomized order. The scores for each parameter ranged from 1 to a maximum of 5, where 1 indicated worst quality and the highest score (5) represented best quality.

### 2.6. Fat Extraction

Fat from ground shortbread cookies (50 g) after their baking and storage (1, 2, and 3 months) was extracted by shaking (Elpan, water bath shaker type 357, Elpin-Plus, Lubawa, Poland) at ambient temperature (21 °C) for 40 min with the use of *n*-hexane. After filtration and separation of the fat, the solvent was removed by evaporation under reduced pressure using a rotary evaporator (Rotavapor R-215, Büchi Labortechnik, Switzerland) at 40 °C. The fat obtained was frozen (−21 °C) until further use. Chemical analysis of the fat extracted from the cookies consisted in determining peroxide value (PV), *p*-anisidine value (*p*-AV), specific UV extinctions (K_232_ and K_268_ values), and antioxidant activity (DPPH assay).

### 2.7. Chemical Analysis

The peroxide and *p*-anisidine values and specific UV extinctions were determined according to ISO standard methods (3960:2009, 6885:2008, 3656:2011, respectively) [29,30,31].

The antioxidant activity of fat samples was determined using DPPH radicals as described by Kozłowska et al. [32,33]. Briefly, 50 mg of each fat sample was dissolved in 3 mL of ethyl acetate. Then, 1 mL of a sample was withdrawn and diluted with 2.75 mL of ethyl acetate and 0.25 mL of a freshly prepared DPPH solution (1 mM) was added. The samples were vigorously mixed for 10 s in a vortex and stored for 20 min at room temperature in the dark. Absorbance was measured at 515 nm using a UV/Vis spectrophotometer (Model 8500, Techcomp, HongKong). The results were expressed as Trolox equivalent antioxidant capacity using a Trolox calibration curve (µmol TEAC/g of oil).

### 2.8. Statistical Analysis

A one-way analysis of variance (ANOVA) and Tukey’s test were used to establish the significance of differences between the means at *p* < 0.05. The statistical analysis was carried out with Statgraphics plus 4.0 package (Statistical Graphics Corp., The Plains, VA, USA).

## 3. Results and Discussion

### 3.1. Color Measurement

Color is such a very important factor in the appearance of shortbread cookies that it affects the choice of products by consumers. Results from instrumental color measurement of the cookies without synthetic and natural antioxidants (the control sample) and those containing BHA and herbal extracts at the level of 0.02, 0.1, and 0.2% are listed in Table 1. Significant differences in L* (lightness), a* (redness), and b* (yellowness) of the cookies without antioxidants and of the cookies enriched with BHA and hyssop, lemon balm, and nettle extracts were observed. The lightness values of the cookies with the added hyssop and lemon balm extracts at concentrations of 0.02 and 0.1% were higher than those of the control sample and the samples containing BHA. The presence of nettle extracts in the cookies caused a decrease in L* values in comparison to the control sample. This kind of cookie was of a darker color than the control sample. Generally, cookies with lower additions of the herbal extracts were characterized by greater lightness than samples with higher additions of the extracts [34,35]. A similar trend has been reported by Balestra et al. [36], where fortification of bread with a low percentage of ginger powder caused L* values for bread crumb to become higher, in effect, these samples were lighter in color. L* values in whole wheat bread also decreased with the addition of various plant materials such as defatted *Cephalaria syriaca* flour, rosehip, and malt flour [37]. This darkness could be due to dark color of the plant materials added, especially when the dark red rosehip material was used. In our study, the lowest L* value was measured for the cookies containing 0.2% of nettle extract (59.03). The nettle extract was also characterized by the darkest color among all the extracts used in the preparation of the shortbread cookies.

The a* and b* values, with all measurements above zero, confirmed that red tone dominated over green and yellow over blue in all the cookies. The highest a* and b* color values were obtained for the sample without additives. The intensity of the red tone for the cookies containing herbal extracts differed regarding the concentration of extracts added. The cookies enriched with a lower amount of lemon balm and nettle extracts were characterized by a greater redness than the cookies with the addition of more plant material. In turn, the presence of hyssop extract at the highest concentration produced the highest a* value. The cookies supplemented with BHA indicated a much higher intensity of the red hue than the cookies with the addition of lemon balm and nettle extracts (0.1 and 0.2%) but showed a lower yellowness (b*) than the samples containing 0.2% lemon balm extract, 0.02% hyssop extract, and nettle extracts at concentrations of 0.1 and 0.2%. Our data showed that decreasing concentrations of lemon balm and nettle extracts reduced b* values with the exception of the cookies with the addition of hyssop extracts. Intensity of the yellow tone for cookies fortified with the hyssop extracts was the highest when this extract was used at a concentration of 0.02% and it was similar to that determined for the control sample. However, the application of hyssop and rosemary extracts as additives for meat processing showed that the b* values were higher than for the control sample (deionized water), so rosemary and hyssop extracts clearly had a protective effect on the b* coordinate [18]. In turn, b* parameter showed statistically lower values in cupcakes containing lemon balm extract rich in rosmarinic acid than in the remaining formulations. It may indicate that this particular type of cupcake might be less visually attractive [38]. In our study, L* and a* values for the cookies enriched with nettle extract (0.2%) were the lowest compared to their counterpart fortified with hyssop and lemon balm extracts and with BHA. A similar tendency was also observed in Latocha and Stasiak [39], in which effect of nettle water extract on lipid oxidation and color of cooked pork sausage was investigated.

### 3.2. Sensory Evaluation

**Figure 1:**
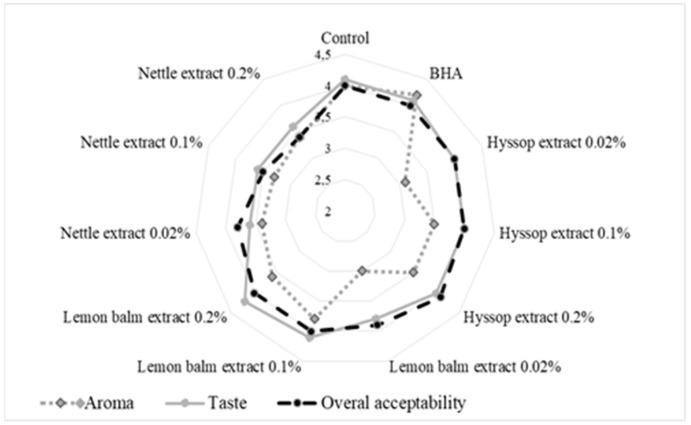
Sensory properties of shortbread cookies enriched with herbal extracts and BHA.

Figure 1 presents results of sensory analysis of the cookies carried out directly after baking by a trained panel of 10 members. The cookies were evaluated for three sensory attributes such as aroma, taste, and overall acceptability.

It was noticed that the highest scores for all the sensory attributes were obtained for cookies without antioxidants, cookies prepared with BHA and 0.1 and 0.2% lemon balm extract. However, the cookies with the addition of nettle extracts received lower scores than the control, BHA, hyssop and lemon balm extracts containing cookies. The aroma of the sample enriched with BHA was the most preferable to the panelists, followed by the control sample and the cookies prepared with lemon balm extracts (0.1 and 0.2%, respectively), hyssop extracts (0.1 and 0.2%, respectively), nettle extracts, and finally the cookies with hyssop and lemon balm extracts at the lowest used concentration (0.02%). In terms of taste and overall acceptability, almost all the tested cookies were accepted by the panelists except those fortified with nettle extracts, although it should be emphasized that mean scores did not differ significantly (*p* < 0.5). Incorporation of hyssop and lemon balm extracts did not have significant influence on overall acceptability of the cookies. This kind of cookies had similar mean acceptability values to the control sample and the sample with BHA addition. In our studies, only the cookies treated with natural antioxidants extracted from nettle, especially at 0.2% level, received lower scores than the control, BHA and other herbal extracts containing cookies.

The biscuits prepared with chloroform extract of Garcinia and with curcumin (95% pure) were also well accepted in terms of color, taste and texture and were comparable to the control biscuits and biscuits enriched with BHA [40]. It was observed that at 10% addition of jering seed flour, the cookies were well accepted and not significantly different from the control sample in terms of color, taste, and overall acceptability [41]. However, Riyazi and Asefi [42] showed that patatoes fried for 48 h in rapeseed oil with methanolic extracts of *Urtica dioica* leaves at the level of 100 ppm were more tasty and generally acceptable as French fries than the samples fried in oil without synthetic antioxidants or containing 100 ppm TBHQ. They also indicated that increasing concentration of nettle extract in rapeseed oil reduced its impact on the score of taste and overall acceptability of the French fries. The results obtained by Belščak-Cvitanović et al. [43] showed that addition of nettle extract to chocolates decreased their sensation of sweetness and escalated their astringency and bitterness which may be regarded as unfavorable by consumers. They also indicated that addition of concentrated nettle extract decreased the scores of all sensory properties of chocolates, whereas addition of freeze dried nettle extract augmented herbal aroma intensity compared to plain chocolate. In Davidov-Pardo et al. [44], cookies without grape seed extract addition were sweeter than enriched cookies. Bitterness of phenolic compounds present in the extracts probably reduced perceived sweetness of the enriched cookies in the same way that sweetness could mask the bitter flavor of the grape seed extract. The overall sensory quality scores of the (control) sausage with water and with dried leaves of nettle were statistically significantly lower than those for samples containing 300 and 600 ppm *U. dioica* extract [39]. The flavor and total acceptability of hamburger patties with lemon balm powder were also highly preferable because the fresh and sweet taste of lemon balm removed the aroma of beef and pork by acting positively on the patties [45].

### 3.3. Chemical Analysis

#### 3.3.1. Changes in Peroxide (PV) and *p*-Anisidine (*p*-AnV) Values

Changes in fat quality parameters such as PV and *p*-AnV values were evaluated after fat extraction from the cookies directly after baking and during of storage at room temperature. The results are presented in Table 2 and Table 3. The PV (Table 2) and *p*-AnV (Table 3) values for fat extracted from all the studied samples ranged from 0.99 to 6.73 meq O_2_/kg and from 2.81 to 7.89, respectively. The content of primary lipid oxidation products (hydroperoxides), determined by PV measurement, was the lowest in fat samples extracted from the cookies enriched with BHA both after baking (at zero time, 0.99 meq O_2_/kg) and during 3 months of storage (1.43 meq O_2_/kg). In the case of fat from the control sample without the antioxidants addition, slow formation of hydroperoxides was also observed whose content increased gradually until the end of the storage time. The addition of herbal extracts as natural antioxidants showed that after baking the PV values for the fat samples were higher than for fat obtained from the control sample. Then, PV in all the fat samples extracted from the cookies fortified with hyssop and lemon balm extracts increased to reach maximum values after one month of storage. After 2 months of the storage, the content of hydroperoxides decreased and a trend of PV reduction was observed up to the end of storage. After these cookies were stored for 3 months, their PV values were similar to those determined directly after baking. It was also noticed that fat extracted from the cookies enriched with 0.02% of hyssop extract contained a lower amount of primary lipid oxidation products than the control sample after 3 months of storage. However, when a higher concentration of hyssop extract was added to the cookies, higher PV values were determined as well. A similar tendency was observed when nettle extracts were added to the cookies. In turn, peroxide values of the samples to which 0.2% lemon balm extract had been added were lower compared to the cookies including 0.02% of lemon balm extract. These results showed that the presence in cookies of BHA and hyssop extract at the level of 0.02% contribute to better protection of lipids against oxidation or delay oxidation.

The PV method is the most commonly used to determine peroxides formed from fatty acids oxidation [34]. Unsaturated fatty acids are particularly susceptible to oxidation, especially when subjected to thermal treatment. In the present study (Table 4), linoleic acid (C18:2, n-9; 40.52%) was the dominant fatty acid in the fatty acid composition of fat used to prepare the shortbread cookies. Additionally, eleven other fatty acids were identified, including such major fatty acids as oleic acid (C18:1, n-9; 29.18%), palmitic acid (C16:0; 21.01%), linolenic acid (C18:3, n-3; 4.68%), and stearic acid (C18:0; 3.36%). Total content of saturated fatty acids in the analyzed fat was 24.93% and of unsaturated fatty acids 75.07%. Among the unsaturated fatty acids, 45.20% of PUFAs were identified. In addition, 0.49% of trans isomer of C18:1 was found. Unstable hydroperoxides may degrade to secondary lipid oxidation products responsible for deterioration of organoleptic properties of food rich in fat. Their content may be expressed by *p*-AnV values. The changes in the *p*-AnV values for all the studied fat samples occurred gradually and slowly during 3 months of storage. Generally, increasing *p*-anisidine values were observed. They were higher in fat samples extracted from the cookies prepared with 0.02 and 0.1% addition of lemon balm extracts than in the remaining samples. The lowest *p*-AnV was detected in samples containing BHA and lemon balm extract at the level of 0.2%. They increased from 2.81 and 3.63 (after baking) to 3.94 and 5.05 (during 3 months of storage), respectively. The control sample exhibited the highest *p*-AnV (except two samples containing lemon balm extracts) throughout the storage period (from 5.32 to 6.73). These results are in agreement with those presented by Kozlowska et al. [46], who reported that cookies also treated with BHA and green tea extract at a concentration of 1% caused a significant reduction of *p*-AnV in the lipid fraction compared to the control sample from baking to the end of storage.

Soleimani et al. [47] reported that a water extract of hyssop was able to reduce the oxidation rate of soybean oil under conditions of the oven test at 70 °C. All samples containing from 200 to 1000 ppm of hyssop extracts were more stable than the control when assessed by peroxide changes, but PV values were lower than BHA used at the concentration of 200 ppm. On the basis of PV assay, Babović et al. [6] determined efficiency of rosemary, sage, thyme, and hyssop extracts as natural antioxidants in stabilization of sunflower oil. They found that the effect of hyssop antioxidant fraction was close to the control sample but weaker in samples enriched with other extracts because of the absence of carnosic acid which may contribute to a stronger antioxidant activity of rosemary and sage extracts. According to our observation, also Buta et al. [48] showed that inhibitory effects of lemon balm extract against primary oxidation of lipids depended on the concentration at which the extract was used. The PV values decreased with increasing concentrations of the lemon balm extract added to refined sunflower oil free from additives. Addition of lemon balm extract to corn oil also showed that its higher content affects slower oxidation of oil [49]. However, corn oil containing 200 ppm of BHA suppressed oxidation more effectively than samples including lemon balm extract at 200, 400, 800, and 1600 ppm. Presence of phenolic compounds in nettle extract influences its antioxidant effect, but significantly less so than plants from the Lamiaceae family such as lemon balm and hyssop extracts [50]. In our studies, the incorporation of nettle extract into cookies caused significant increases of PV values during 3 months of storage, especially when the highest dose was used. Aqueous nettle extract inhibited peroxidation in linolenic acid emulsion dose-dependently and more distinctly than α-tocopherol [51]. When it was used as a popular supplement for pigs, increasing lightness of meat and stabilized meat color for 6 months of storage at −20 °C were observed. Moreover, it slightly improved meat oxidative stability during frozen storage [52]. Supplementing of broilers with chokeberry pomace and nettle was without effect on performance, carcass weight and composition of the broiler compared with the control sample. Nettle also proved unsuitable as a dietary antioxidant in the applied form and concentration [53].

#### 3.3.2. Specific Extinction Coefficients Measurement

The K values measured at 232 and 268 nm give information about the degree of fat oxidative deterioration. K_232_ is highly associated with the amount of conjugated dienes as a consequence of primary oxidation of polyunsaturated fatty acids. However, K_268_ is more correlated to the presence of secondary oxidation compounds such as conjugated trienes and carbonyl compounds [54]. Changes in K_232_ and K_268_ values are shown in Table 5. For all the fat samples studied, both the values increased with the storage time, but formation of conjugated trienes was slower and in many cases negligible, especially after 2 months of the storage. The content of conjugated dienes was the lowest in the fat samples obtained by extraction of cookies enriched with 0.02% of BHA and hyssop extract directly after baking and after 3 months of storage, and was correlated with PV values. It was also noticed that low PV values for the control sample during the entire storage period did not indicate a similar trend regarding K_232_ values determined up to the end of their storage. K_232_ values for the control sample were lower than for the fat samples from the cookies enriched with BHA and herbs extracts after one month of storage. Generally, the presence of BHA and herbal extracts insignificantly delays formation of conjugated dienes in supplemented samples but did not depend on the concentration at which the herbal extracts were used. After baking, K_232_ values decreased with an increased percentage of lemon balm and nettle extracts (from 0.02 to 0.2%) but increased when hyssop extracts were used. This trend was not similar to that observed in the first and second months of storage. At that time, a better effect of hyssop and lemon balm extracts on slower formation of conjugated dienes was observed when these extracts at 1% concentration were incorporated into the cookies. In Cagdas and Kumcuoglu [55], the highest conjugated dienes concentrations were recorded in all lipids samples extracted from precooked and frozen chicken nuggets treated with grape seed powder in the second month of their storage. However, it was also observed that the conjugated dienes values decreased with a rising percentage of grape seed powder. The presence of green and roasted coffee extracts in two different confectionary products (filled chocolate and cookies baked at 200 °C) reduced the content of conjugated dienes in the tested products [56]. Their content was generally higher than the content of conjugated trienes and this observation was in agreement with our results. Stabilization of soybean oil with plant extracts from Labiateae family also delayed formation of conjugated dienes and trienes in the supplemented samples [57]. Effectiveness of this plant material is often related to a high content of flavonoids and phenolic compounds responsible for antioxidant activity. Among synthethic antioxidants, *tert*-butylhydroquinone (TBHQ) was more effective than BHA and served as the strongest antioxidant in borage and evening primrose oil TAG [58].

#### 3.3.3. Antioxidant Activity

The antioxidant activity of fat samples extracted from the cookies was evaluated using the DPPH assay and the results were expressed as Trolox equivalent antioxidant capacity. All the studied fat samples, except for fat extracted from the cookies enriched with 0.1% hyssop extract, showed a higher ability to scavenge DPPH radicals than the control sample during the entire storage of the samples (Table 6). It was also observed that the ability of fat to quench DPPH radicals decreased during the storage period and was not linearly dependent on the concentration of herbal extracts added to the cookies. After baking, the fat samples extracted from the cookies enriched with 0.02 and 0.1% nettle extracts, 0.2% lemon balm and hyssop extracts exhibited a stronger antioxidant activity than the fat sample with BHA. After 3 months of storage, the fat samples obtained from the cookies fortified with BHA, 0.02% hyssop and nettle extracts and 0.2% lemon balm extract presented the highest antioxidant activity among the studied samples. The addition of 0.2% lemon balm extract to the cookies caused an almost two times higher ability of the received fat samples to scavenge free radicals directly after the end of the baking process than of the control sample. However, after 3 months of the storage, the determined DPPH values were lower but still, in the presence of BHA, almost two times higher compared to the untreated samples. After 3 months of the storage, the use of 0.2% lemon balm extract as a natural additive resulted in an about 1.6 times higher antioxidant capacity of the fat sample compared to the sample without an enrichment. The samples enriched with BHA, 0.2% lemon balm extract and 0.02% nettle extract were characterized by the highest DPPH values until the end of storage. What is more, the incorporation of BHA into the cookies improved oxidative stability of the fat samples determined by the lowest PV and *p*-AnV values. Kozłowska et al. [46] observed that the samples fortified with BHA and 1% green tea extracts exhibited a greater resistance to oxidation than those from cookies without additives. In turn, rosemary extract was a more effective antioxidant in the rapeseed oil TAG than *α*-, *β*-, *δ*-tocopherols alone and BHT [59]. The time required to reduce DPPH radical scavenging capacity by 50% was increased from 15 h to 39 h when sesame seed extract was added to olive oil [60]. The addition of “Vitalplant” extract improved antioxidant activity and oxidative stability of the cookies because of synergistic effects of the extract ingredients [61].

## 4. Conclusions

The addition of herbal extracts to shortbread cookies may improve their sensory properties and extend shelf life by protecting fat against oxidation. The present study showed that cookies enriched with lower amount of herbal extracts were characterized by greater lightness values than samples with higher additions of the extracts. Their fortification with synthetic antioxidant (BHA) and lemon balm extracts at 0.1 and 0.2% concentrations recorded the highest scores of all sensory attributes relative to other enriched cookie samples. The use of 0.02% hyssop extract and BHA resulted in lower peroxide values of extracted fat at three months of storage compared to the control sample. Increases of *p*-anisidine values were observed in all fat samples but were lower compared to fat extracted from the cookies without additives, except those containing 0.02 and 0.1% lemon balm extracts. Presence of BHA and herbal extracts insignificantly delayed formation of conjugated dienes in the supplemented samples but it did not depend on concentrations at which the herbal extracts were used. Fat extracted from the cookies enriched with a synthetic antioxidant and herbal extracts, except 0.1% hyssop extract, exhibited a greater ability to scavenge of DPPH radicals than the control sample did. These data showed that the use of lemon balm and hyssop extracts in formulation of shortbread cookies may positively affect quality of the cookies and may be attractive to consumers. However, fat samples extracted from cookies enriched with BHA were less susceptible to oxidation compared to the samples containing herbal extracts. BHA was still a better inhibitor of fat oxidation.

## Figures and Tables

**Table 1:** Color characteristics of shortbread cookies enriched with herbal extracts and butylated hydroxyanisole (BHA) ^1^.

Sample		Parameter		
		L*	a*	b*
**Control**		65.28 ± 1.34 ^e^	5.45 ± 0.68 ^h^	24.50 ± 1.05 ^f,g^
**BHA**	**0.02%**	63.83 ± 2.12 ^c^	4.25 ± 0.89 ^e^	23.76 ± 1.12 ^d^
**Hyssop extract**	**0.02%**	66.81 ± 1.12 ^g^	4.15 ± 1.11 ^e^	24.38 ± 1.12 ^f^
	**0.1%**	66.45 ± 1.45 ^f,g^	3.30 ± 0.87 ^d^	23.08 ± 1.32 ^b^
	**0.2%**	63.26 ± 1.54 ^b^	4.45 ± 1.34 ^f^	22.91 ± 1.34 ^b^
**Lemon balm extract**	**0.02%**	66.31 ± 1.23 ^f^	2.84 ± 0.99 ^c^	22.66 ± 0.78 ^a^
	**0.1%**	66.64 ± 1.45 ^f,g^	2.83 ± 1.23 ^c^	23.58 ± 0.67 ^d^
	**0.2%**	64.46 ± 1.57 ^d^	2.68 ± 1.09 ^c^	25.47 ± 0.87 ^h^
**Nettle extract**	**0.02%**	64.82 ± 1.78 ^d^	4.68 ± 0.95 ^g^	23.36 ± 1.14 ^c^
	**0.1%**	63.57 ± 1.85 ^b,c^	1.59 ± 0.76 ^b^	24.08 ± 1.25 ^e^
	**0.2%**	59.03 ± 1.87 ^a^	1.37 ± 1.09 ^a^	24.60 ± 1.28 ^g^

^1^ Data are displayed as mean ± SD. Mean values marked by the different lower-case superscripts letters within a column denote statistically significant differences (*p* < 0.05).

**Table 2:** Peroxide values (PV) of fat extracted from shortbread cookies enriched with herbal extracts and BHA after baking and during the storage at room temperature ^1^.

Sample		PV [meq O_2_/kg]			
		Storage Time (Months)			
		0	1	2	3
**Control**		1.06 ± 0.02 ^B,a^	1.13 ± 0.01 ^B,b^	1.26 ± 0.02 ^A,c^	1.66 ± 0.03 ^C,d^
**BHA**	**0.02%**	0.99 ± 0.04 ^A,a^	1.03 ± 0.02 ^A,b^	1.25 ± 0.04 ^A,c^	1.43 ± 0.06 ^A,d^
**Hyssop Extract**	**0.02%**	1.47 ± 0.12 ^C,a^	2.11 ± 0.09 ^C,d^	1.94 ± 0.06 ^B,c^	1.49 ± 0.08 ^B,b^
	**0.1%**	2.05 ± 0.03 ^D,a^	3.52 ± 0.11 ^E,d^	2.98 ± 0.10 ^C,c^	2.18 ± 0.12 ^D,b^
	**0.2%**	3.57 ± 0.07 ^G,a^	4.98 ± 0.17 ^H,d^	4.23 ± 0.21 ^F,c^	3.73 ± 0.16 ^H,b^
**Lemon Balm Extract**	**0.02%**	3.87 ± 0.25 ^I,a^	5.12 ± 0.15 ^I,d^	4.43 ± 0.22 ^G,c^	3.95 ± 0.14 ^J,b^
	**0.1%**	3.42 ± 0.17 ^F,b^	4.78 ± 0.14 ^H,d^	3.82 ± 0.17 ^E,c^	3.39 ± 0.14 ^F,a^
	**0.2%**	2.78 ± 0.09 ^E,b^	3.94 ± 0.07 ^F,d^	2.98 ± 0.11 ^C,c^	2.76 ± 0.12 ^E,a^
**Nettle Extract**	**0.02%**	3.67 ± 0.11 ^H,d^	3.45 ± 0.08 ^D,c^	3.33 ± 0.13 ^D,a^	3.43 ± 0.17 ^G,b^
	**0.1%**	4.98 ± 0.29 ^J,d^	4.75 ± 0.13 ^G,c^	4.53 ± 0.16 ^H,a^	4.61 ± 0.21 ^J,b^
	**0.2%**	6.13 ± 0.21 ^K,b^	5.89 ± 0.17 ^J,a^	6.28 ± 0.21 ^J,c^	6.73 ± 0.26 ^K,d^

^1^ Data are displayed as mean ± SD. Mean values marked by the different lower-case (a–d) superscripts letters within a row denote statistically significant differences (*p* < 0.05). Value marked by the different upper-case superscripts letters (A–K) within a column denote statistically significant differences (*p* < 0.05).

**Table 3:** *p*-Anisidine values (*p*-AnV) of fat extracted from shortbread cookies enriched with herbal extracts and BHA after baking and during the storage at room temperature ^1^.

Sample		*p*-AnV			
		Storage Time (Months)			
		0	1	2	3
**Control**		5.32 ± 0.22 ^I,a^	5.40 ± 0.19 ^I,b^	5.93 ± 0.32 ^I,c^	6.73 ± 0.39 ^J,d^
**BHA**	**0.02%**	2.81 ± 0.08 ^A,a^	2.96 ± 0.11 ^A,b^	3.46 ± 0.21 ^A,c^	3.94 ± 0.05 ^A,d^
**Hyssop Extract**	**0.02%**	5.18 ± 0.16 ^H,a^	5.35 ± 0.49 ^H,b^	5.78 ± 0.36 ^H,d^	5.77 ± 0.28 ^F,c^
	**0.1%**	4.60 ± 0.07 ^G,a^	4.64 ± 0.21 ^F,b^	4.95 ± 0.21 ^F,c^	5.45 ± 0.22 ^C,d^
	**0.2%**	4.18 ± 0.11 ^C,a^	4.25 ± 0.18 ^D,b^	4.38 ± 0.19 ^C,c^	5.63 ± 0.26 ^E,d^
**Lemon Balm Extract**	**0.02%**	5.63 ± 0.21 ^J,a^	5.68 ± 0.45 ^J,b^	7.04 ± 0.52 ^K,c^	7.89 ± 0.44 ^K,d^
	**0.1%**	5.77 ± 0.14 ^K,a^	5.79 ± 0.34 ^K,b^	6.89 ± 0.57 ^J,c^	6.98 ± 0.34 ^I,d^
	**0.2%**	3.63 ± 0.10 ^B,a^	4.09 ± 0.27 ^B,b^	4.46 ± 0.71 ^D,c^	5.05 ± 0.32 ^B,d^
**Nettle Extract**	**0.02%**	4.19 ± 0.13 ^D,a^	4.21 ± 0.18 ^C,b^	4.36 ± 0.16 ^B,c^	5.55 ± 0.27 ^D,d^
	**0.1%**	4.59 ± 0.19 ^F,a^	4.88 ± 0.13 ^G,b^	5.07 ± 0.19 ^G,c^	5.86 ± 0.25 ^G,d^
	**0.2%**	4.27 ± 0.21 ^E,a^	4.29 ± 0.17 ^E,a^	4.50 ± 0.16 ^E,b^	6.02 ± 0.29 ^H,c^

^1^ Data are displayed as mean ± SD. Mean values marked by the different lower-case (a–d) superscripts letters within a row denote statistically significant differences (*p* < 0.05). Value marked by the different upper-case superscripts letters (A–K) within a column denote statistically significant differences (*p* < 0.05).

**Table 4:** Fatty acids composition of fat used to the cookies preparation ^1^.

Fatty Acids	Area [%]	RT [min]
**C12:0** (Lauric acid)	0.06 ± 0.01 ^a^	8.065
**C14:0** (Myristic acid)	0.23 ± 0.02 ^a^	8.421
**C16:0** (Palmitic acid)	21.01 ± 0.04 ^d^	8.862
**C16:1** (Palmitoleic acid)	0.08 ± 0.01 ^a^	8.934
**C17:0** (Margaric acid)	0.05 ± 0.01 ^a^	9.605
**C18:0** (Stearic acid)	3.36 ± 0.02 ^b^	10.388
**C18:1, n-9c** (Oleic acid)	29.18 ± 0.03 ^e^	10.453
**C18:1, n-9t** (Elaidic acid)	0.49 ± 0.01 ^a^	10.771
**C18:2, n-6** (Linoleic acid)	40.52 ± 0.05 ^f^	11.239
**C18:3, n-3** (α-Linolenic acid)	4.68 ± 0.03 ^c^	11.881
**C20:0** (Arachidic acid)	0.22 ± 0.01 ^a^	12.464
**C20:1** (Gondoic acid)	0.12 ± 0.01 ^a^	12.668
**∑ SFA**	24.93 ± 0.04	
**∑ MUFA**	29.87 ± 0.06	
**∑ PUFA**	45.20 ± 0.08	

^1^ Data are displayed as mean ± SD. Mean values marked by the different lower-case superscripts letters within a column denote statistically significant differences (*p* < 0.05).

**Table 5:** Conjugated dienes and trienes values expressed as specific extinction coefficients (K_232_ and K_268_) in fat extracted from shortbread cookies enriched with herbal extracts and BHA after baking and during the storage at room temperature. ^1^ Data are displayed as mean ± SD. Mean values marked by the different lower-case (a–d) superscripts letters within a row denote statistically significant differences (*p* < 0.05). Value marked by the different upper-case superscripts letters (A–K) within a column denote statistically significant differences (*p* < 0.05).

Sample		Storage Time (Months)							
		0		1		2		3	
		K_232_	K_268_	K_232_	K_268_	K_232_	K_268_	K_232_	K_268_
**Control**		1.44 ± 0.02 ^C,a^	0.78 ± 0.01 ^D,E,a^	1.89 ± 0.03 ^G,b^	0.79 ± 0.01 ^E,a,b^	2.03 ± 0.02 ^I,c^	0.81 ± 0.01 ^E,b,c^	2.98 ± 0.03 ^I,d^	0.82 ± 0.02 ^D,c^
**BHA**	**0.02%**	1.09 ± 0.01 ^A,a^	0.60 ± 0.02 ^A,a^	1.24 ± 0.02 ^A,b^	0.65 ± 0.01 ^A,b^	1.47 ± 0.02 ^A,c^	0.67 ±0,01 ^A,c^	1.78 ± 0.03 ^A,d^	0.69 ± 0.02 ^A,d^
**Hyssop Extract**	**0.02%**	1.42 ± 0.00 ^B,a^	0.77 ± 0.02 ^C,D,E,a^	1.84 ± 0.02 ^F,b^	0.82 ± 0.01 ^F,b^	2.09 ± 0.02 ^J,c^	0.84 ± 0.01 ^F,c^	2.49 ± 0.02 ^D,d^	0.88 ± 0.02 ^E,d^
	**0.1%**	1.53 ± 0.01 ^G,a^	0.75 ± 0.02 ^C,a^	1.69 ± 0.02 ^C,D,b^	0.76 ± 0.01 ^C,D,a,b^	1.86 ± 0.01 ^F,c^	0.77 ± 0.01 ^C,D,a,b^	2.62 ± 0.03 ^F,d^	0.78 ± 0.02 ^B,C,b^
	**0.2%**	1.55 ± 0.02 ^I,a^	0.76 ± 0.02 ^C,D,a^	1.78 ± 0.01 ^E,b^	0.78 ± 0.02 ^D,E,a,b^	1.94 ± 0.02 ^G,c^	0.79 ± 0.01 ^D,E,b^	2.78 ± 0.03 ^G,d^	0.81 ± 0.02 ^D,c^
**Lemon Balm Extract**	**0.02%**	1.63 ± 0.01 ^J,a^	0.82 ± 0.01 ^F,a^	1.75 ± 0.01 ^E,b^	0.83 ± 0.02 ^F,G,a^	1.88 ± 0.02 ^F,c^	0.84 ± 0.02 ^F,a^	2.93 ± 0.03 ^H,d^	0.88 ± 0.01 ^E,b^
	**0.1%**	1.49 ± 0.02 ^D,a^	0.83 ± 0.02 ^F,a^	1.67 ± 0.01 ^C,b^	0.85 ± 0.01 ^G,a,b^	1.79 ± 0.02 ^E,c^	0.87 ± 0.02 ^G,b^	2.55 ± 0.02 ^E,d^	0.87 ± 0.01 ^E,b^
	**0.2%**	1.50 ± 0.00 ^E,a^	0.79 ± 0.02 ^E,a^	1.91 ± 0.01 ^G,b^	0.79 ± 0.02 ^E,a^	1.98 ± 0.02 ^H,c^	0.80 ± 0.02 ^E,a^	2.51 ± 0.03 ^D,d^	0.82 ± 0.01 ^D,b^
**Nettle Extract**	**0.02%**	1.64 ± 0.01 ^K,a^	0.78 ± 0.02 ^D,E,a^	1.69 ± 0.02 ^C,D,b^	0.78 ± 0.02 ^D,E,a^	1.70 ± 0.01 ^C,b^	0.79 ± 0.02 ^D,E,a^	3.29 ± 0.03 ^J,c^	0.80 ± 0.02 ^C,D,a^
	**0.1%**	1.54 ± 0.02 ^H,a^	0.75 ± 0.01 ^C,a^	1.71 ± 0.01 ^D,b^	0.75 ± 0.02 ^B,C,a^	1.76 ± 0.02 ^D,c^	0.76 ± 0.02 ^B,C,a^	2.44 ± 0.02 ^C,d^	0.76 ± 0.02 ^B,a^
	**0.2%**	1.51 ± 0.01 ^F,a^	0.72 ± 0.02 ^B,a^	1.55 ± 0.01 ^B,b^	0.73 ± 0.02 ^B,a^	1.57 ± 0.02 ^B,c^	0.74 ± 0.01 ^B,a^	2.08 ± 0.01 ^B,d^	0.78 ± 0.01 ^B,C,b^

**Table 6:** Antioxidant activity of fat extracted from shortbread cookies enriched with herbal extracts and BHA after baking and during the storage at room temperature measured by DPPH method ^1^.

Sample		TEAC (µmol/g fat)			
		Storage Time (months)			
		0	1	2	3
**Control**		0.91 ± 0.09 ^B,d^	0.82 ± 0.07 ^A,c^	0.78 ± 0.06 ^B,b^	0.71 ± 0.05 ^B,a^
**BHA**	**0.02%**	1.52 ± 0.14 ^F,d^	1.47 ± 0.15 ^G,c^	1.40 ± 0.12 ^I,b^	1.32 ± 0.10 ^I,a^
**Hyssop Extract**	**0.02%**	1.49 ± 0.13 ^F,d^	1.39 ± 0.12 ^F,c^	1.12 ± 0.10 ^D,b^	1.06 ± 0.09 ^F,a^
	**0.1%**	0.85 ± 0.07 ^A,d^	0.81 ± 0.07 ^A,c^	0.68 ± 0.07 ^A,b^	0.54 ± 0.04 ^A,a^
	**0.2%**	1.56 ± 0.16 ^G,d^	1.16 ± 0.12 ^B,c^	1.15 ± 0.12 ^E,b^	0.96 ± 0.07 ^D,a^
**Lemon Balm Extract**	**0.02%**	1.31 ± 0.12 ^D,d^	1.18 ± 0.10 ^B,c^	0.90 ± 0.07 ^C,b^	0.82 ± 0.06 ^C,a^
	**0.1%**	1.15 ± 0.11 ^C,b^	1.25 ± 0.11 ^C,d^	1.17 ± 0.12 ^E,c^	1.01 ± 0.09 ^E,a^
	**0.2%**	1.86 ± 0.16 ^H,d^	1.35 ± 0.12 ^E,c^	1.15 ± 0.11 ^E,b^	1.11 ± 0.10 ^G,a^
**Nettle Extract**	**0.02%**	1.56 ± 0.14 ^G,d^	1.30 ± 0.12 ^D,c^	1.22 ± 0.10 ^F,b^	1.18 ± 0.11 ^H,a^
	**0.1%**	1.84 ± 0.16 ^H,b^	1.31 ± 0.11 ^D,a^	1.30 ± 0.12 ^H,a^	1.03 ± 0.08 ^E,a^
	**0.2%**	1.36 ± 0.12 ^E,d^	1.33 ± 0.12 ^D,e^	1.27 ± 0.12 ^G,b^	0.84 ± 0.06 ^C,a^

^1^ Data are displayed as mean ± SD. Mean values marked by the different lower-case (a–d) superscripts letters within a row denote statistically significant differences (*p* < 0.05). Value marked by the different upper-case superscripts letters (A–I) within a column denote statistically significant differences (*p* < 0.05).
